# Circulating Total and Active Metalloproteinase-9 and Tissue Inhibitor of Metalloproteinases-1 in Patients with Systemic Lupus Erythomatosus

**DOI:** 10.1155/MI/2006/17898

**Published:** 2006-02-06

**Authors:** Ewa Robak, Agnieszka Wierzbowska, Magdalena Chmiela, Liliana Kulczycka, Anna Sysa-Jędrejowska, Tadeusz Robak

**Affiliations:** ^1^Department of Dermatology and Venerology, Medical University of Lodz, Krzemieniecka 5, 94-017 Lodz, Poland; ^2^Department of Hematology, Medical University of Lodz and Copernicus Memorial Hospital in Lodz, Pabianicka 62, 93-513 Lodz, Poland; ^3^Department of Immunology and Infectious Biology, University of Lodz, Banacha 12/16, 90-237 Lodz, Poland

## Abstract

We investigated the serum concentration of total metalloproteinase-9 (tMPP-9), active MMP-9 (aMMP-9), and tissue inhibitor of metalloproteinase-1 (TIMP-1) in a group of 41 patients with SLE and 20 healthy controls. Serum levels of tMMP-9 and TIMP-1 were assessed by an enzyme-linked immunosorbent assay (ELISA) and aMMP-9 by fluorometric assay. The tMMP-9 level was lower in SLE patients (mean 262 ng/mL) than in healthy volunteers (mean 325 ng/mL) (*P* = .048). Similarly, aMMP-9 level was lower in SLE patients (mean 121 ng/mL) than in control group (mean 169 ng/mL) (*P* = .0355) and lower in active SLE (mean 54 ng/mL) than in inactive disease (mean 99 ng/mL) (*P* = .033). TIMP-1 level was also lower in SLE patients (mean 181 ng/mL) than in control group (mean 233 ng/mL) (*P* = .004). In SLE patients, a positive correlation was found between tMMP-9 and aMMP-9 (ρ = 0.568; *P* = .001). We also found a positive correlation of tMMP-9 and TIMP-1 with VEGF concentrations (ρ = 0.450, *P* = .005 and ρ = 0.387; *P* = .018, resp). tMMP-9, aMMP-9, and TIMP-1 serum levels are lower in SLE patients than in healthy control group.

## INTRODUCTION

Systemic lupus erythematosus (SLE) is an autoimmune disease characterized by B-cell hyperactivity, the formation of pathogenic autoantibodies, and highly varied clinical manifestations [1, 2]. Among organs and systems targeted in this disease the skin, joints, kidneys, nervous system, serosal surfaces, and blood cells are the most common sites of involvement.

The involvement of angiogenesis and angiogenic factors in pathogenesis of SLE has been recently suggested [3–6]. Angiogenesis is a multistep process in which new blood vessels grow from existing vessels [7]. Extracellular matrix remodeling, endothelial cell migration and proliferation, capillary differentiation and anastomosis are the sequential steps required for angiogenesis. A family of pro- and antiagiogenic factors tightly regulates this process. A large number of cytokines have been shown to stimulate angiogenesis, including vascular endothelial growth factor (VEGF).

In addition to growth factors and cytokines, extracellular matrix components such as matrix metalloproteinases (MMP) have been implicated in angiogenesis [8]. Among them MMP-2 and MMP-9, also called gelatinases A and B, are reported to cleave a wide variety of substrates, although their primary substrates are considered to be gelatin. MMP-9 is also involved in inflammation and immune system dysfunctions [9, 10]. MMP-9 originates from monocytes, macrophages, neutrophils, keratinocytes, fibroblasts, endothelial cells, and various tumor cells. It is secreted in the form of latent 92 kd zymogens that need to undergo proteolytic and autocatalytic activation to 82 kd form [11].

MMPs are inhibited by specific proteins—the tissue inhibitors of metalloproteinases (TIMP) [12]. TIMP-1 is one of the four natural inhibitors of MMPs. It is a 28.5 kd glycoprotein that forms a noncovalent 1:1 stoichiometric complex with MMPs, thereby inhibiting the proteolytic activity of these enezymes [12]. TIMP-1 binds with high affinity to the inactive pro-MMP-9 forming a complex in which TIMP-1 retains its ability to inhibit the activity of another MMP via its N-terminal domain. Some physiological functions of TIMP-1 are linked to the functions of MMP and an improper balance in their productions may have a role in several diseases including cancer and rheumatoid arthritis [13]. Moreover TIMP-1 inhibits apoptosis of B-cells [14].

MMPs and their inhibitors may play a role in pathogenesis of SLE and other connective tissue diseases [9, 14–20]. In the present study we measured the serum concentrations of total and active MMP-9 as well as TIMP-1 in patients with SLE. The serum levels of these proteins were correlated with disease activity and some clinical and laboratory parameters. To the best of our knowledge a simultaneous evaluation of these proteins in patients with SLE has not been investigated to date.

## PATIENTS AND METHODS

The study group consisted of 41 patients with SLE (38 females and 3 males) and 20 sex- and age-matched healthy volunteers. The median age of SLE patients was 40.5 years (range 19–73) and 38 years (range 16–68) in control group. The diagnosis of SLE was based on the revised criteria of the American Rheumatism Associaton [21]. Twenty-five patients were treated with steroids and/or other immunosuppressive agents. In all patients the activity of the disease was determined according to the systemic lupus activity measure (SLAM) scale [22]. Each patient was examined on two separate occasions, 2–4 weeks apart. The system of SLAM includes 24 clinical manifestations and eight laboratory parameters. The maximum score in this system is 84 points. In our group of patients, the number of points ranged from 9–25. In the present study we considered a score of 0–15 points as inactive disease and score over 15 points as active diseases. By this definition, active disease was found in 19 patients while 22 patients had inactive disease. The clinical and laboratory features of SLE patients are presented in Table 1.

Each person underwent a thorough physical evaluation by one of the authors (E. Robak). The patients with SLE and controls showed no clinical signs of infection or neoplastic disease and received neither antibiotics nor other medications for at least 4 weeks prior to blood donation. This project was performed in accordance with the Helsinki Declaration.

An informed consent was obtained from all patients participating in the study. The project was approved by the local Ethics Committee (Medical University of Lodz, no RNN 25/05/KE).

### Laboratory tests

On the day of blood sampling for MMP-9 and TIMP-1 the following laboratory parameters were analysed: complete blood cell count, erythrocyte sedimentation rate (ESR), urinalysis, blood urea, nitrogen and creatinine levels, fibrinogen level, partial thromboplastin time (PTT), liver function tests (GOT, GPT, bilirubin), immunoglobulins (IgG, IgA, IgM) complement (C3, C4), and anti-DNA antibodies (ANA). The lupus band test (LBT) was also determined. Chest X-rays and abdominal ultrasonography were performed in all patients.

### Serum sampling and MMP-9, TIMP-1, and VEGF determination

Venous blood samples were collected at the time of clinical assessment in pyrogen free tubes, allowed to dot at −4°C for 1 hour and centrifuged at 2000 for 10 minutes. The serum obtained was divided into aliquots and stored at −25°C until assayed for MMP-9, TIMP-1, and VEGF.

The detection of the serum levels of total MMP-9, TIMP-1, and VEGF was performed using ELISA sandwich kits employing human anti-MMP-9, anti-TIMP-1, and anti-VEGF antibodies (R&D Systems, Inc, Minneapolis, MN) using horse radish peroxidase detection in accordance with the manufacturer's instructions. The absorption was read at 492 nm. The appropriate recombinant human cytokine was used to generate the standard curve for each assay. The concentration of cytokines in the samples was determined by the interpolation from the standard curve. Standards as well as samples were assayed as duplicates and the interassay variations were shown to be within the range given by the manufacturer. This procedure has been described precisely in our previous work [23, 24]. The sensitivity limit for VEGF was 5.0 pg/mL, tMMP-9–0.156 ng/mL, and TIMP-1–0.08 ng/mL.

The detection of the serum aMMP-9 level was performed using fluorometric assay designed to quantitatively measure enzyme activity (R&D Systems, Inc). The Fluorokine E Active MMP-9 kit is designed to measure the levels of endogenous active MMP-9. All manufacturer's instructions were followed. As suggested 100-fold dilution of the serum with Calibrator Diluent RD 5–24 was used. Standard MMP-9 samples were activated during the assay with the addition of AMPA (*p*-aminophenyl mercuric acetable). A standard curve was generated for each set of samples assayed. The sensitivity limit for this assay was 0.005 ng/mL. The results of all proteins' measurements were presented in units recommended by ELISA kit producer.

### Statistical analysis

For the statistical analysis of the data the range of measured variable (minimum-maximum), the mean arithmetic value (*x*), the median (Me), and the standard deviation (SD) were calculated. The Shapiro-Wilk test was used to evaluate the distribution. The mean values were compared using the Kruskal-Wallis test and Mann-Whitney's test. The correlation between features was evaluated using the Spearman rank coefficient ρ. Comparisons and correlations were considered significant when *P* < .05.

## RESULTS

### The serum levels of tMMP-9, aMMP-9, TIMP-1, and VEGF

In the group of 41 SLE patients, 19 were with active and 22 with inactive disease according to the Liang et al [22] scoring system. The serum concentrations of total and active MMP-9 and TIMP-1 were detectable in all SLE patients and in healthy volunteers. The results are presented in Table 2. The tMMP-9 level was higher (mean 262 ng/mL) than aMMP-9 (mean 121 ng/mL) in SLE patients (*P* = .001) and in control group (325 ng/mL and 169 ng/mL, resp, *P* = .001). The level tMMP-9 was lower in SLE patients (mean 262 ng/mL) than in healthy volunteers (mean 325 ng/mL) (*P* = .023). The concentration of tMMP-9 was lower in active SLE (mean 182.6 ng/mL) than in inactive disease (mean 333 ng/mL) (*P* = .048). However, the levels of tMMP-9 in inactive SLE and healthy persons were similar (*P* > .05). Similarly, aMMP-9 level was lower in SLE patients (mean 121 ng/mL) than in control group (mean 169 ng/mL) (*P* = .035). Moreover, a lower concentration of aMMP-9 was in active SLE than in inactive disease (mean 54 ng/mL and 99 ng/mL, resp, *P* = .033). TIMP-1 level was also lower in SLE patients (mean 181 ng/mL) than in control group (mean 233 ng/mL) (*P* = .004). The levels of TIMP-1 in active and ininactive disease were similar (*P* > .05). Moreover, we found no statistically significant correlation between SLE activity score according to the SLAM index and the level of tMMP-9 (ρ = 0.223; *P* > .05), aMMP-9 (ρ = 0.222, *P* > .05), and TIMP-1 (ρ = 0.111; *P* > .05).

The concentrations of VEGF in SLE patients and in control group were similar in this study (*P* > .05) (Table 2).

### The correlations between investigated proteins

**Figure 1:**
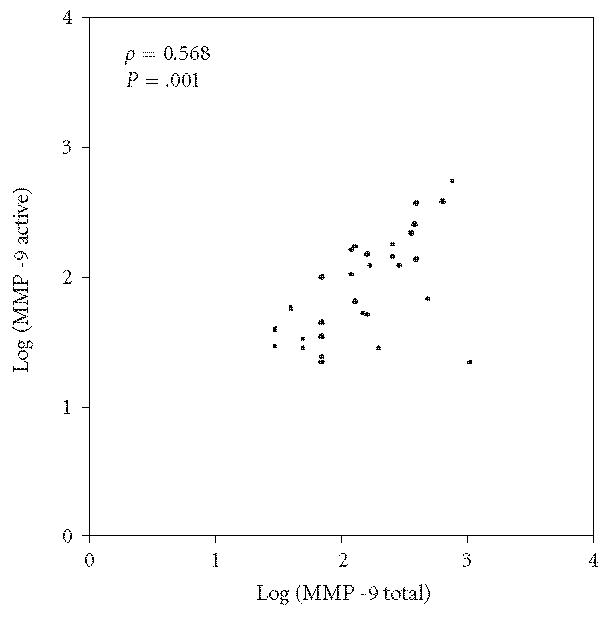
Correlations between total and active MMP-9 in SLE patients.

**Figure 2:**
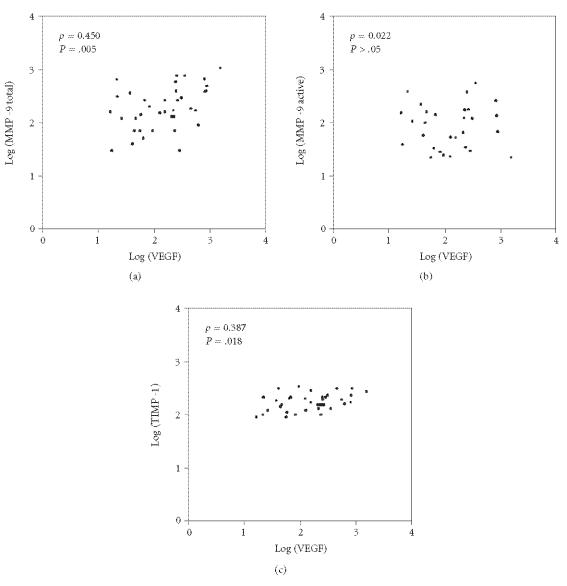
Correlations between VEGF and MMP-9 total, MMP-9 active, and TIMP-1 serum levels.

A positive correlation was found between tMMP-9 and aMMP-9 in SLE patients (ρ = 0.568; *P* = .001) (Figure 1). However, the correlations of aMMP-9 with TIMP-1 and tMMP-9 with TIMP-1 were not statistically significant. We analyzed the correlation between serum levels of tMMP-9, aMMP-9, and TIMP-1 with VEGF (Figure 2). We found a positive correlation of VEGF with tMMP-9 (ρ = 0.450; *P* = .005) and with TIMP-1 (ρ = 0.387; *P* = 0.018), but not with aMMP-9 (ρ = 0.022, *P* > .05).

### The correlations of investigated proteins with clinical and laboratory parameters

In this study we compared the serum levels of tMMP-9, aMMP-9, and TIMP-1 with several clinical and laboratory symptoms of the disease. However the differences were not statistically significant, except for a higher level of tMMP-9 in patients without antinuclear antibodies (440 ng/mL) than in the patients with titer of antinuclear antibodies > 160 (145 ng/mL) (*P* = .003). The concentrations of these proteins were also similar in the patients treated and untreated with steroids and/or cytotoxic agents (data not shown).

## DISCUSSION

The aim of our study was to assess serum concentrations of total and active MMP-9 and its tissue inhibitor (TIMP-1) in patients with active and inactive SLE and in healthy volunteers. Detectable concentrations of these factors were found either in all patients with SLE and all healthy volunteers. However, the concentrations of tMMP-9 and aMMP-9 were unexpectedly lower in patients with SLE as compared with control groups. Moreover, lower concentration of tMMP-9 was detected in patients with active SLE as compared with patients with inactive disease.

The sparse data from literature concerning serum MMP-9 concentration in patients with SLE are heterogeneous. Faber-Elmann et al [19] found the increased activity of MMP-9 in serum of patients with SLE. However, Chinese authors, similarly to our research, found lower levels of MMP-9 in patients with SLE in comparison with healthy subjects [25]. Besides, they observed lower concentration of MMP-9 in serum of patients with active SLE as compared with inactive disease, similarly as in our patients. These scientists did not report, however, what type of MMP-9 was determined. In our research we did not find any correlation between the SLE activity according to the SLAM scale and tMMP-9 or aMMP-9 concentrations. These observations are consistent with results described by Faber-Elmann et al [19], who also did not demonstrate any correlation between serum MMP-9 level and the Systemic Lupus Erythematosus Disease Activity Index.

A significant part of our research was to assess a correlation between the presence or absence of clinical and laboratory SLE symptoms and serum tMMP-9 and aMMP-9 levels. This research did not show any statistically significant correlations between concentrations of these factors and the presence of any clinical or laboratory symptoms, except the presence of antinuclear antibodies. However, a lack of statistical significance can result from the small number of patients in the given groups. Makowski and Ramsby [15] examined a correlation between MMP-9 concentration and anti-sDNA or anti-dsDNA levels showing reverse correlation with anti-dsDNA, which is a specific marker of SLE. Similarly, Liu et al [25] observed lower concentration of MMP in patients with lupus nephritis as compared with patients with SLE without renal impairment. These observations are consistent in patients with active SLE as compared with patients with inactive disease.

Lower concentration of MMP-9 in serum of patients with SLE, especially with active disease detected either in our research or in studies performed by other authors bring to mind some interpretative difficulties. It was found that the peripheral blood mononuclear cells (PBMC) in patients with SLE form and secrete more MMP-9 than their counterparts in healthy volunteers [9]. Moreover, the most increased pro-MMP-9 activity inside the PBMCs was identified for relapsed SLE subgroup. It can be assumed that in SLE, more MMP-9 is transported from blood to the lupoid tissues, especially blood vessels in the more active SLE patients. Mawrin et al [17] showed that in patients with SLE and peripheral neuropathy, MMP-3 and MMP-9 can be detected in vessel walls of nerves when in healthy subjects they were not found. The authors suggest that up-regulation of MMP-3 and MMP-9 within the vessels may be responsible for vascular damage seen in SLE.

To date even less attention has been paid to the role of TIMP-1 in patients with SLE in comparison with MMP-9. According to our knowledge our research is the first in which this factor was determined in serum of patients with SLE. In our studies TIMP-1 concentration, similarly as MMP-9, was lower in patients with SLE than in healthy volunteers. Matache et al [9] did not find any significant differences in the formation of this protein by leukocytes in patients with SLE and in healthy subjects, however higher amounts were formed in leukocytes of patients with more active SLE. However, we did not find significant differences in this cytokine concentration in patients with active and inactive SLE, and in patients with various clinical and laboratory symptoms of this disease. These results are different from those obtained by Toubi et al [20] in patients with scleroderma. In this pathology of connective tissue, the concentration of TIMP-1 was higher as compared with healthy volunteers and correlated with the severity of scleroderma. Similar correlations between serum TIMP-1 concentrations were observed by Tayebjee et al [26], in patients with angiographically proven peripheral arterial disease, in which TIMP-1 concentration also correlated with the severity of clinical symptoms.

In our studies we did not show any correlations between serum TIMP-1 concentration and tMMP-9 and aMMP-9 levels. However, this correlation was observed by some authors in healthy controls [27]. In our studies we showed the positive correlation between concentrations of tMMP, TIMP-1, and VEGF. These results can be at least partially explained by the fact that the above-mentioned agents are prerequisite factors for angiogenesis.

To summarize, we have concluded that in patients with SLE, serum tMMP-9, aMMP-9, and TIMP-1 levels are lower as compared with healthy volunteers. The levels of these factors do not correlate with the activity of SLE according to the SLAM classification, or with the presence of particular clinical and laboratory symptoms of SLE. Moreover, we showed a positive correlation between tMMP-9, TIMP-1, and VEGF concentrations in sera of patients with SLE. Lower concentrations of MMP-9 and TIMP-1 in patients with SLE can result from the accumulation of these factors in the inflamed blood vessels and tissues.

## Figures and Tables

**Table 1:** Clinical and laboratory characteristics of SLE patients.

Symptoms	Number of patients	(%)

Total	41	100%
Age (years)		
Mean (range)	40.5 (19–73)	—
Sex (male/female)	3/38	7%/93%
Active/inactive	19/22	46%/54%
Arthritis	34	83%
Skin symptoms	32	78%
Reticuloendothelial system involvement	23	56%
Renal disorder (kreatinine > 1.3 mg/dL)	4	40%
Neurologic symptoms	27	66%
Antinuclear antibodies titer > 160	38	93%
dsDNA antibodies	6	15%
Anemia (Hb < 12 g/dL)	18	44%
Leukopenia (WBC < 3.5 × 10^9^/L)	14	34%
Thrombocytopenia (platelets < 150 × 10^9^/L)	12	29%
C reactive protein (> 5.99 mg/L)	4	10%
Raised ESR (> 25 mm/h)	20	49%
*C*_3_ < 0.9	12	29%
*C*_4_ < 0.1	5	12%
Immunosuppressive treatment with steroid and/or cytotoxic	25	61%
agents during the study		

**Table 2:** Serum levels of VEGF, total and active metalloproteinase-9 (MMP-9), and TIMP-1 in patients with SLE and control group (mean values of VEGF in pg/mL, metalloproteinase-9, and TIMP-1 in ng/mL).

Factor	All SLE	Active SLE	Inactive SLE	Control group	Statistically significant comparison	
	*n* = 41	*n* = 19	*n* = 22	*n* = 20		
	(a)	(b)	(c)	(d)

VEGF $\bar{x}$ ± *s*	285 ± 233	253 ± 234	312 ± 404	208 ± 163	—	
MMP-9 total $\bar{x}$ ± *s*	262 ± 242	183 ± 164	333 ± 280	325 ± 168	(a)–(b)	*P* = .023
					(b)–(c)–(d)	*P* = .014
(b)–(c)					*P* = .048
(b)–(d)					*P* < .001

MMP-9 active $\bar{x}$ ± *s*	121 ± 123	90 ± 71	147 ± 151	169 ± 104	(a)–(d)	*P* = .035
					(b)–(c)–(d)	*P* = .033
					(b)–(d)	*P* = .016

TIMP-1 *x ± s*	181 ± 67	193 ± 72	170 ± 61	233 ± 64	(a)–(d)	*P* = .004
					(b)–(c)–(d)	*P* = .038
					(c)–(d)	*P* = .002
